# Why is Medical Dosimetry a profession only in the United States and what does this mean for Medical Physicists worldwide?

**DOI:** 10.1002/acm2.13362

**Published:** 2021-07-16

**Authors:** Sasha Graham, Michael Mills

**Affiliations:** ^1^ Department of Radiation Oncology the University of Texas M.D. Anderson Cancer Center Houston TX USA; ^2^ Department of Radiation Oncology University of Louisville Louisville KY USA

One of the curiosities of Radiation Oncology is the difference between the professional practice models inside and outside the United States. In this Editorial, we want to focus on the historical development of the Medical Dosimetry profession within the United States, and the almost complete absence of full‐time professional planners outside. Given the future likely reality of Artificial Intelligence (AI) generated plans, what is the role of Medical Dosimetrists in the future? Is the current Medical Dosimetry professional model and the associated infrastructure an effort worth exporting to other nations? This idea is worth considering, because if full‐time planners can provide services at higher quality and lower cost than currently exists outside the United States, ultimately this benefits the patient. If the model is to be exported, the ones to accomplish this are the younger members of the American Association of Medical Dosimetrists (AAMD) and the Medical Dosimetry community. The co‐author Sasha Graham, a Medical Dosimetrist under 30 years old, offers her perspective.

“A Medical Dosimetrist is a vital part of the radiation oncology team. The highly specialized training they receive ensures that a patient will be treated with a high quality of care. As a Medical Dosimetrist, I see a great benefit in having a specialized role when providing cancer care to patients. I was given the opportunity in school to have access to different forms of planning, different treatment planning systems, and mentors that provided in‐depth training. We are given the tools to learn how effectively to use the treatment planning system and our mentors provide us with real time feedback. This allows us to become great planners during the time we are in school and to be ready to enter the workforce.

It is interesting to me that most other countries do not have a Medical Dosimetrist on staff. A Medical Dosimetrist’s role is being completed by a physicist or radiation therapist. This brings up a lot of questions about how effective is the system without a Medical Dosimetrist and what is the quality level of the treatment that these patients are getting? Medical Physicists have many roles and many duties to fulfill as part of the radiation oncology team. By also being required to do the treatment plans, does this have an influence on other parts of their job? I truly believe that with a trained Medical Dosimetrist, the quality of treatment plans would be far greater and would have a large impact on the patient. A trained Medical Dosimetrist would know how to manipulate the treatment planning system and in turn, know how effectively to push dose off organs and important structures while treating the cancer. Not only would this increase the quality of care for the patient but this would provide Medical Physicists with adequate time to complete their other job duties. I believe that the level of QA provided by the Medical Physicist would increase with a Medical Dosimetrist on staff because more people would be reviewing and checking the plan before the patient even gets treated. Overall, I believe an increase in patient safety and a decrease in errors would be seen by allowing a Medical Dosimetrist to do the treatment planning.

If a Medical Physicist who is performing the treatment planning tasks was shown the benefits of having a Medical Dosimetrist on staff, I believe they would agree that it was worthwhile. By allowing Physicists, Dosimetrists, and Radiation Therapists to focus on their skill set, more patients could be treated and a higher quality of care can be provided. Change is hard for most people but I do not think you can deny the benefits of having a Medical Dosimetrist on staff. I think by allowing Medical Physicists to see our work model in the United States and how effective the radiation oncology team works together; they would be willing to move towards updating their work structure.

I find myself becoming very passionate about this subject because I truly believe that the Medical Dosimetry career matters and has an impact on patient’s lives. That is why I joined the field and I love what I do. If we could be providing a higher level of care at any center, community, or country then I would hope we would work to make improvements where we can. I hope to see Medical Dosimetry roles start to take precedence in other countries but I know that it will take efforts by me and other members of my field.”

The history of the AAMD is summarized in this excellent article by Christopher J. Moore: https://www.meddos.org/article/S0958‐3947(15)00029‐1/fulltext. Far from being imposed from the top‐down, the profession seemed to rise organically, and emerged during the 1970s and early 1980s. It went through the typical growing pains of any profession. It should be pointed out that the creation and maintenance of a respected academic journal, *Medical Dosimetry*, is very rare for a medical profession consisting largely of those with Associate and Bachelor’s degrees. The profession is to be commended for the success of the *Medical Dosimetry* journal. The wealth of education and training materials, the quality of the academic programs, and the supporting infrastructure of the AAMD, including the *Medical Dosimetry* journal, puts it in a unique position to export the Medical Dosimetry professional model to the rest of the world, if there is a community of will to accomplish this goal.

There are, however, some significant barriers to exporting Medical Dosimetry curriculum and clinical training:

- Although the programs may be US based, there may be issues with translation of curriculum
- Clinical sites would need to be accredited by an international accrediting organization
- The Joint Review Committee on Education in Radiologic Technology (JRCERT) is a US‐only accrediting organization. This is stated in their Charter. It is likely another method of accrediting international clinical Medical Dosimetry training sites would need to be identified
- In some countries, the Medical Physicists are not paid well and are very protective of their unique duties. Consequently, there may be resistance to the presence of Medical Dosimetrists
- In many countries, the national government would need to be supportive of and approve a new training program in Medical Dosimetry
- Given the state of development of machine‐learning and deep‐learning tools, one could argue that we are only a few years away from having consistent treatment planning results of acceptable quality performed mostly by algorithms and at a much lower cost. In that eventuality, what future staff would take control of this process?
- Given the AI planning eventuality, what is the future role of Medical Dosimetry in the United States?

If the compensation of the full‐time Medical Dosimetrist is closer to the salary of an Radiation Therapy Technologist than it is to that of a Medical Physicist, the resulting plans are likely to be more cost‐efficient if performed by Medical Dosimetrists rather than Medical Physicists. Also, it may be stated with confidence that one has only to attend a regional or national meeting of the AAMD to experience the very high level of sophistication and professionality practiced by full‐time Medical Dosimetrists in the United States. It is probable this knowledge needs or will need to exist in the community of professionals responsible for the AI planning procedures. AI planning may take some time to emerge fully; 20 years would not be too short an estimate. Radiation oncology planning is performed everywhere on the planet; therefore, this business model should hold everywhere because Medical Dosimetrists lower the transaction costs of providing plans of high quality and safety. However, in many nations, this training would also be applicable for Medical Physicists, so the design of the international training model could include this consideration and flexibility.

Given the almost certain eventuality of AI‐based planning, it is nevertheless difficult to provide clear focus as to the role of Medical Dosimetry over the next 20 years. If past experience is any guide, the emergence of paradigm‐shifting technologies may produce the demand for more labor rather than less. It is certainly possible that the special skills needed to provide those last manual tweaks to AI‐generated plans may become very valuable skills indeed, whether provided by Medical Dosimetrists or Medical Physicists. It may also be that AI will replace some of what US Medical Dosimetrists are currently doing, but it is likely they will end up doing other things: QA, tweaking the AI, and possibly acting as physician extenders, answering technical questions from patients and their families. Regardless, full‐time planners will have a significant role to play in the future of radiation oncology, worldwide.

